# Molecular Circuits of Resolution in the Eye

**DOI:** 10.1100/tsw.2010.99

**Published:** 2010-06-01

**Authors:** Elvira L. Liclican, Karsten Gronert

**Affiliations:** Vision Science Program, School of Optometry, University of California, Berkeley, CA, USA

**Keywords:** inflammatory resolution, ω-3 polyunsaturated fatty acids, lipoxin A4, resolvins, neuroprotectin D1, lipoxygenase

## Abstract

Lipid autacoids have well-established key roles in physiology and pathophysiology. Eicosanoids derived from ω-6 arachidonic acid (AA) have long been recognized for their roles in cardiovascular and renal functions, and vascular tone, as well as regulating inflammatory and immune functions. It is now appreciated that AA is a substrate for generating lipid mediators with anti-inflammatory and proresolving properties, namely lipoxins (i.e., LXA4), which are an integral component for the successful execution of beneficial and essential acute inflammatory responses. In addition to AA, the ω-3 polyunsaturated fatty acids (PUFAs) eicosapentaenoic acid (EPA) and docosahexaenoic acid (DHA) also serve as substrates to generate potent and protective autacoids, such as resolvins and neuroprotectin (i.e., NPD1), respectively. These ω-3–derived signals may mediate the remarkable protective action of essential dietary ω-3 PUFAs. Formation and bioactivity of lipid mediators in the eye are relatively unexplored and of considerable interest, as the eye contains highly specialized tissues, including the transparent avascular and immune-privileged cornea, and the neuro-retina. A rapidly emerging field has identified key biosynthetic enzymes, receptors, and temporally defined endogenous formation of ω-3– and ω-6–derived protective lipid circuits in the eye. Protective endogenous roles of LXA4 and NPD1 have been established utilizing lipidomic analysis, knockout mice, and pharmacological, genetic, and dietary manipulation, providing compelling evidence that these intrinsic lipid autacoid circuits play essential roles in restraining inflammation, promoting wound healing, inhibiting pathological angiogenesis, and providing neuroprotection in the delicate visual axis.

### INTRODUCTION

The eye is one of the primary sense organs and is composed of a posterior outgrowth of the central nervous system, the retina, and an anterior highly modified transparent skin, the avascular cornea. The main function of this unique and specialized organ is to convert light energy into nerve action potentials, a highly conserved process that is dependent on maintaining the refractive properties of the cornea, lens, aqueous humor, and vitreous humor; the formation and reabsorption of fluids that keep intraocular pressure constant; and preserving the function of retinal neurons. In order to preserve function, it is essential for the delicate visual axis to maintain an avascular, transparent, and/or immune-privileged state in several ocular tissues.

Hence, the threat of inflammation is incompatible with good vision, especially in the highly specialized anterior surface (i.e., cornea) and neural tissue (i.e., retina) of the eye. However, the visual axis faces constant inflammatory and immunogenic threats, as the ocular surface is continuously exposed to the shearing stress of eyelid motion, prolonged periods of hypoxia associated with sleep, and, due to its anatomical positioning, environmental pathogens and irritants. In addition, the retina is vulnerable to blood-borne pathogens, oxidative stress, inflammatory mediators, and systemic inflammatory/immune disease, despite its isolation by a well-developed blood tissue barrier. Thus, it is not surprising that the eye contains highly developed circuits to control inflammation, maintain host defense, and promote ordered and rapid wound healing, thereby protecting essential vision[1,2,3,4,5,6,7,8,9].

Although it shares many features with immunity in other tissues, the ocular immune response is atypical. Like other tissues that are extremely vulnerable to inflammation-induced tissue injury, such as the brain and pregnant uterus, the eye has developed key adaptations to minimize collateral tissue injury by restraining immune and inflammatory responses[2,3,4,9,10,11]. A remarkable feature of the eye is the presence of avascular, transparent, and immune-privileged sites, such as the cornea. In particular, the cornea actively maintains an anti-inflammatory and immunosuppressive microenvironment that can accept, sometimes indefinitely, allogeneic grafts without immune rejections and heals rapidly with almost no scarring in response to mild injury while maintaining unwavering host defense[3,4,5,6,9,12,13,14,15,16,17]. Execution of these fundamental and self-resolving processes in the eye is clearly regulated by interdependent circuits and temporally defined arrays of mediators, which remain to be clearly defined. Lipid mediators of inflammation, proliferation, tissue remodeling, and/or angiogenesis have well-appreciated and key roles in physiology and pathophysiology. Compared to long-standing research efforts that have defined lipid autacoid circuits as therapeutic targets, and delineated their complex and pleiotropic molecular mechanisms of action in most tissues/organs, our understanding of lipid mediator circuits in the eye is just beginning to unfold[6,16,18,19,20,21,22,23,24]. An emerging body of work provides strong evidence that protective lipid circuits are critical components of inflammatory resolution, apoptosis, wound healing, and pathological angiogenesis in the eye, which is the focus of this review.

### LIPID AUTACOID CIRCUITS IN THE EYE

Eicosanoids are derived from the essential polyunsaturated fatty acid (PUFA) arachidonic acid (AA, ω-6 C20:4). Following injury, AA is released and can be metabolized in a cell-specific fashion by three families of enzymes, cyclooxygenases (COXs), lipoxygenases (LOXs), and cytochrome P450s (CYP450s), to form lipid autacoids. All three families of enzymes are functionally expressed in the eye as are G protein-coupled receptors for COX-derived prostaglandins (PGs), and LOX-derived lipoxins (LXs) and leukotrienes (LTs), which mediate the actions of these distinct classes of potent lipid autacoids[6,16,19,20,21,25,26].

### Cyclooxygenase (COX)

Early studies have shown that PGs are released during ocular inflammation[27]. However, most studies have focused on the ability of PGs, specifically $PGF_{2\alpha}$, to reduce intraocular pressure[28,29,30]. Thus, the role of PGs in ocular inflammation remains to be fully explored, which is underscored by the fact that despite the therapeutic use of nonsteroidal anti-inflammatory drugs (NSAIDs) for more than 100 years for reducing inflammation and inflammatory hyperalgesia in most tissues, the first ophthalmic topical use of NSAIDs was not until 1986 for maintaining pupil dilation after cataract surgery[31]. Several studies have demonstrated that COX-2 expression and PG formation are up-regulated in models of ocular inflammation[19,32,33,34,35,36]. Moreover, a role for PGs in regulating inflammation in most tissues of the eye is strongly supported by studies that have employed NSAIDs, PG analogs, and PG receptor knockout mice[29,37,38,39,40]. COX-derived PGs exert diverse and sometimes opposing bioactivity, which is receptor- and cell type–specific, as well as temporally defined. In this regard, research efforts in the eye are only in the early stages. Specifically, $PGE_2$, acting through four distinct receptors (EP1, EP2, EP3, and EP4) that are expressed in the eye[41], can induce vascular, pro-, or anti-inflammatory effects and biosynthetic pathways for proresolving lipid autacoids (i.e., LX), as well as regulate fibrosis[6,42,43,44,45,46,47,48]. However, the endogenous role of $PGE_2$ in the eye remains to be elucidated. It is now appreciated that PGs also have important roles in the active resolution of inflammation[42,49,50]. In particular, $PGD_2$ and its cyclopentenone PG breakdown product, 15-deoxyΔ-$PGJ_2$ (Fig. 1), control the balance of cytokines and chemokines that regulate leukocyte influx during acute inflammation, as well as macrophage efflux to draining lymph nodes, leading to resolution[49,51]. Further, 15-deoxyΔ-$PGJ_2$ can inhibit activation of NF-κB and up-regulate expression of the cytoprotective and anti-inflammatory heme-oxygenase-1[49,52,53]. $PGF_{2\alpha}$, which is the primary treatment option for lowering ocular hypertension[28], has also been reported to regulate inflammatory resolution in a carageenin-induced pleurisy model, along with $PGD_2$ and 15-deoxyΔ-$PGJ_2$[54,55,56]. However, the endogenous formation and bioactivity of these protective PG circuits have not been explored in the eye.

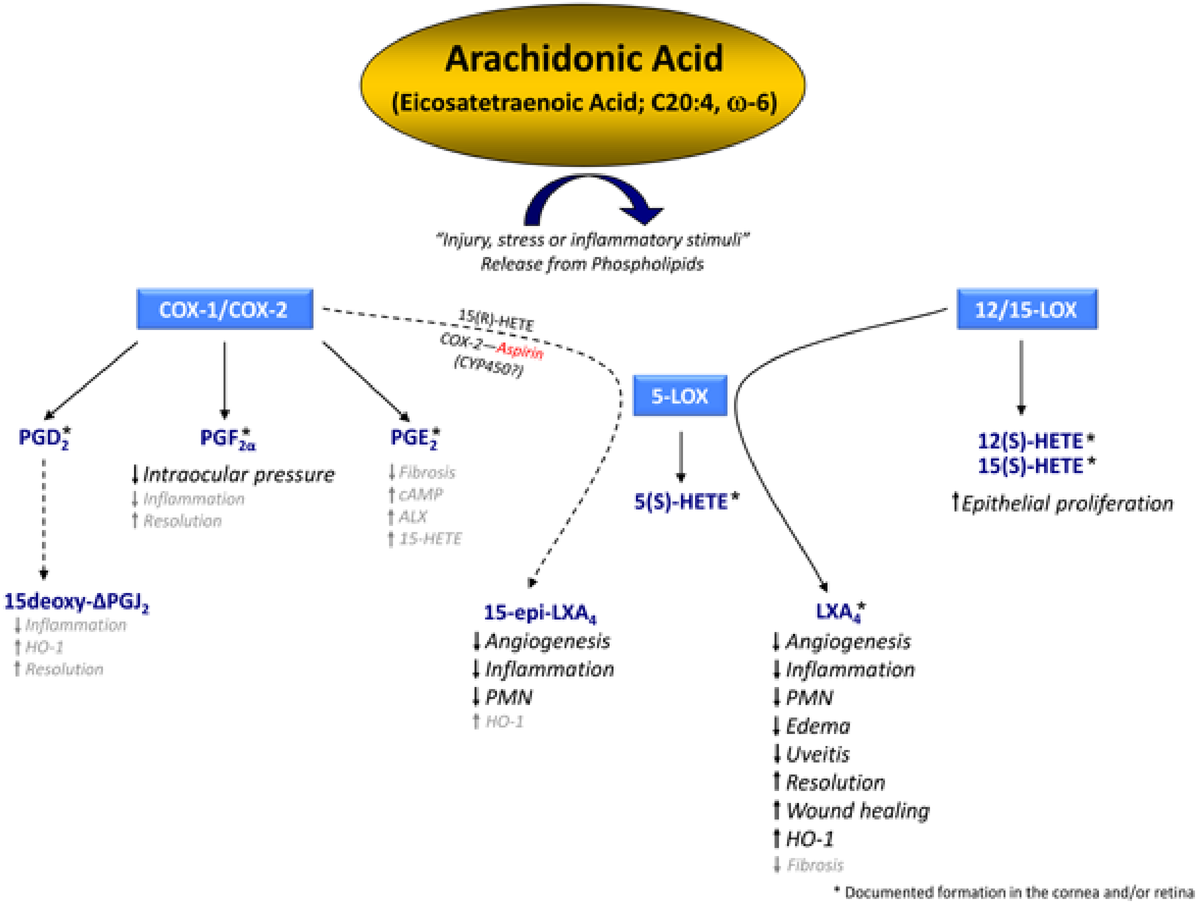

**FIGURE 1.** Protective AA (ω-6)–derived circuits in ocular inflammation. Following injury, stress, or inflammatory stimuli, AA (ω-6) is released from phospholipid pools and can be metabolized by COX or LOX to form lipid autacoids with anti-inflammatory and proresolving properties. Black font denotes established bioactivity in the eye, gray font denotes potential bioactivity not yet explored in the eye.

### Lipoxygenase (LOX)

Lipoxygenases (5-, 12-, and 15-LOX) initially produce fatty acid hydroxyperoxides by stereoselective insertion of molecular oxygen at specific carbons in PUFAs to form 5(S)-, 12(S)-, or 15(S)-

hydroperoxides (H(p)ETE), respectively, if AA (eicosatetraenoic acid) is the substrate. These unstable hydroperoxides are rapidly hydrolyzed to form monohydroxy fatty acids (HETE), or can be converted to epoxides (5-LOX, 15-LOX) and/or further metabolized to pro- or anti-inflammatory lipid mediators, LT or LX, respectively, in the same cell or by cell-cell interactions. Several studies have demonstrated LOX activity in the retina[57,58,59,60], lens[61,62,63], and cornea[16,64,65,66,67] as evidenced by species-specific formation of 15-HETE, 12-HETE, or 5-HETE that was attenuated by general LOX inhibitors. There is considerable species-specific variation in the expression and function of 12-LOX and 15-LOX enzymes. However, enzymatic, gene, and protein data obtained from dog, mouse, rat, rabbit, and human tissues clearly indicate that 12/15-LOX (human, ALOX15; murine, Alox15) is a predominant enzyme in the retina and cornea. RT-PCR analysis and PCR cloning have demonstrated expression of 15-LOX type 1 and 2 (ALOX15 and ALOX15B) in human corneas, whose functional expression is strongly supported by the observation that human corneas and corneal epithelial cells produce one prominent HETE, namely, 15(S)-HETE[65,66,68,69,70]. Monkey corneal epithelial cells also predominantly express 15-LOX[71]. Elucidation of the LOX pathways in the mouse eye has been more complicated, owing to the expression of at least five distinct 12-LOX enzymes in mice. In addition to a 12/15-LOX (Alox15), mice express platelet-type (Alox12) and epidermal-type 12-LOX (Aloxe), which show prominent expression in epithelial cells[72,73,74,75]. mRNA expression of Alox15, Alox12, Aloxe, and 5-LOX (Alox5) has been detected in mouse corneas. However, Alox15 is the most abundant mRNA transcript[76] and evidence from Western blot analysis indicates that platelet-type 12-LOX protein is not expressed in the eye[73]. 12/15-LOX is highly and selectively expressed in the corneal epithelium, as de-epithelialization of the mouse cornea completely abrogated its mRNA expression[76]. Functional expression of both 5-LOX and 12/15-LOX in the mouse cornea is evidenced by endogenous 5-HETE, 15 HETE, as well as $LXA_4$ formation[76], which is significantly impaired in 12/15-LOX–deficient mice[16]. Human retinal pigment epithelial cells express 15-LOX type 1, as its genetic knockdown results in a significant reduction in 15-HETE and $LXA_4$ formation[77], and 12/15-LOX is also expressed in the rat retina[60]. Immunohistochemistry of a whole mouse eye (Fig. 2) provides further evidence for the selective and high expression of 15-LOX, especially in epithelial cells of the cornea, retina, and lens. The endogenous role of the resident 15-LOX circuit in the eye is largely protective, as it is a key enzyme for the generation of the anti-inflammatory and proresolving eicosanoid $LXA_4$.

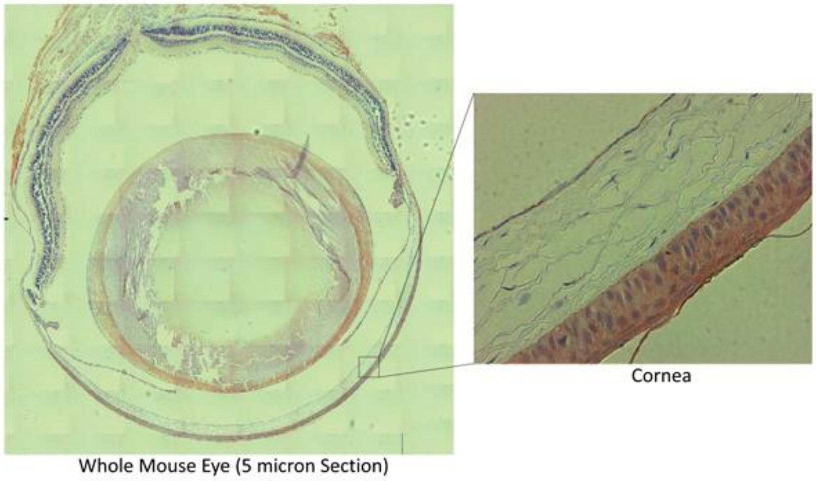

**FIGURE 2.** 12/15-LOX (Alox15) is highly and selectively expressed in epithelial cells of the cornea, retina, and lens. Representative image of a 5-µm section of a healthy, uninjured, whole eye obtained from Balb/c mice, probed with an anti-12/15-LOX antibody, and a magnified section of the cornea.

### Cytochrome P450 (CYP450)

Cytochrome P450 (CYP450) enzymes generate epoxide- and hydroxyeicosanoids, and are ubiquitously expressed throughout the body. They have long been recognized for their roles in xenobiotic metabolism, vascular tone, and cardiovascular and renal functions[78,79,80,81,82,83], but evidence now implicates an emerging role for CYP450 in inflammation[84,85,86,87]. This is of particular interest in the eye, as genetic linkage analysis and mutation studies have linked the CYP1B1 gene to the pathogenesis of glaucoma[88]. Further, CYP2C-derived epoxyeicosatrienoic acids have been implicated in hypoxia-induced retinal angiogenesis[89]. CYP450 has been identified in the corneal epithelium of several animal species, including humans, mice, and rabbits, and studies have established it as a primary corneal epithelial inflammatory pathway in experimental models of ocular surface inflammation[84]. In the cornea, CYP4B1 metabolizes AA to 12(R)-hydroxyeicosatetraenoic acid (12(R)-HETE), which is further metabolized to 12(R)-hydroxyeicosatrienoic acid (12(R)-HETrE). 12(R)-HETrE is a potent angiogenic factor and exerts inflammatory actions, including vasodilation and neutrophil chemotaxis[90]. However, it is important to note that CYP450 enzymes can also promote proresolving pathways in other tissues. Specifically, it has been shown that CYP450 enzymes in some epithelial cells can initiate the formation of a $LXA_4$ isomer, namely 15-epi-$LXA_4$, by generating 15(R)-HETE[91], which resists metabolic inactivation[92]. It remains to be determined if CYP450 enzymes contribute towards endogenous formation of $LXA_4$ in the eye.

### Platelet-Activating Factor (PAF)

Platelet-activating factor (PAF) is a prominent proinflammatory lipid autacoid that is synthesized by a variety of cell types, and exerts diverse physiological and pathological effects[18,19,36,93,94,95]. Its role in inflammatory responses has been extensively investigated in many tissues, and its bioactivity includes microvascular leakage, vasodilation, and activation of several types of inflammatory cells, such as neutrophils, eosinophils, and macrophages[18,19,36,93,94,95]. Its formation has been documented in several tissues of the eye, including the cornea, retina, iris, and ciliary body, and is a key mediator of corneal inflammatory responses[18,19,36]. In addition, PAF impairs re-epithelialization after corneal injury and promotes neovascularization. The effects of PAF are mediated through a G protein-coupled receptor, PAF-R, that activates signals involved in inflammation, wound healing, and apoptosis, and whose corneal expression is up-regulated following injury[18,19,36]. Indeed, the central role of PAF in corneal inflammatory responses is evident by studies that demonstrate that PAF-R antagonists reduce apoptosis, inflammatory cell infiltration, and expression of inflammatory cytokines, as well as prevent the development of keratitis and neovascularization[19,36,96,97].

## LIPID AUTACOID CIRCUITS OF RESOLUTION IN THE EYE

A hallmark response to injury and infection is the recruitment of effector cells of host defense, such as polymorphonuclear leukocytes (PMNs), macrophages, and lymphocytes[8]. However, execution of the vital, highly frequent, and reoccurring inflammatory response necessitates active resolution in order to restore normal tissue function; this is especially critical for the delicate visual axis, as uncontrolled inflammation and disordered wound repair can impair visual acuity and lead to blindness. In this regard, a rapidly evolving field of research has identified distinct classes of endogenous anti-inflammatory and proresolving lipid mediators. An impressive body of work has demonstrated that these endogenous lipid circuits are integral components of an acute inflammatory response, which is dependent on the balanced activation of pro- and anti-inflammatory circuits, controlled wound healing, and active resolution of leukocytes[42,50,98,99,100,101]. Further, emerging evidence places these protective lipid circuits as key components of the ocular inflammatory/reparative response.

### 15-LOX and $LXA_4$ Circuit

Lipoxins (LXs) were the first endogenous lipid mediators with anti-inflammatory and proresolving properties to be discovered[102]. LXs are lipoxygenase-interaction products that are formed from AA during PMN interactions with platelets, endothelial cells, or epithelial cells, and are formed predominantly during the resolution phase of acute inflammation[103,104,105,106]. 12/15-LOX is a key enzyme for their biosynthesis (Fig. 1) and, thus, it is not surprising that the enzyme is highly expressed in epithelial cells and is one of the most prominent inducible genes in human monocytes[107]. An impressive body of work has established that $LXA_4$ is an important mediator of inflammatory resolution. Indeed, several lines of evidence from numerous animal models of acute inflammation, asthma, pathological angiogenesis, host defense, and innate immune responses, as well as human clinical data, indicate that $LXA_4$ and the $LXA_4$ receptor (ALX) constitute an essential protective eicosanoid circuit[103,104,105,106,108].

The bioactivity of $LXA_4$ is mediated via its G protein-coupled receptor, ALX[109]. Importantly, data from receptor transgenic mice and detailed structure-activity studies have established specific ALX receptors in humans (FPRL1) and mice (Fprl1 and Fpr-rs2) as a molecular mechanism for $LXA_4$'s protective *in vivo* actions[109]. $LXA_4$ has cell-specific actions with leukocytes, lymphocytes, and epithelial and endothelial cells, all of which cumulate in reducing the inflammatory response, regulating PMN function, clearing of apoptotic PMNs, and promoting wound healing[50,105,109,110,111,112, 113,114].

In recent years, the essential role of this anti-inflammatory circuit in maintaining an orchestrated and beneficial ocular inflammatory/reparative response has been investigated. A study in 1998 documented the expression of ALX mRNA in human corneas, providing the first evidence for a potential specific bioactivity of $LXA_4$ in the eye[115]. Subsequent studies in mice revealed that $LXA_4$ is produced in the healthy, uninjured mouse cornea[16,76,116]. The fact that the avascular and immune-privileged cornea highly expresses 15-LOX and ALX points toward a central role of this protective lipid circuit in the healthy cornea. In the healthy cornea that contains no leukocytes, 12/15-LOX and ALX are predominantly expressed in epithelial cells, as $LXA_4$ formation and expression of 12/15-LOX and ALX are abrogated by de-epithelialization and restored during epithelial wound healing[16,76]. Hence, in the cornea, $LXA_4$ formation, unlike in most tissues, is not directly dependent on infiltrating leukocytes from the cornea[76].

Direct evidence for a protective role of endogenous $LXA_4$ in the cornea has been obtained through studies in mice deficient in key $LXA_4$ biosynthetic enzymes, namely, 12/15-LOX and 5-LOX[6,16,76,116]. Mice with a targeted deletion of 12/15-LOX display delayed epithelial wound healing, which correlates with impaired endogenous corneal $LXA_4$ formation[76]. Pharmacological amplification of this circuit by topical treatment with $LXA_4$ increased the rate of wound healing, reduced tissue damage following thermal cauterization, and rescued delayed wound healing in 12/15-LOX knockout mice. The protective effects of $LXA_4$ following de-epithelialization were not associated with inhibiting PMN infiltration[76], which is distinct from $LXA_4$'s established mechanism of action to inhibit PMN recruitment in several *in vivo* models of acute inflammation[105]. Hence, in the context of a mild and self-resolving epithelial injury, recruitment of PMNs to the injured cornea is a beneficial protective response that likely depends on a resident $LXA_4$ circuit to restrain activation of these primary and precarious effector cells. Indeed, although early studies indicate that PMNs have no significant or a negative impact on wound healing[117], several studies in the eye now demonstrate that inhibition of PMN infiltration into the cornea is associated with impaired epithelial wound healing[118,119,120,121,122]. Topical treatment with $LXA_4$ also markedly reduced the formation of KC (mouse homolog to human IL-8) and monocyte chemoattractant protein-1[16,76], chemokines that are prominent in acute inflammatory lesions of the eye and keratitis[123,124,125]. Thus, in addition to accelerating epithelial wound healing, $LXA_4$ inhibits the formation of selective epithelial inflammatory markers in the cornea.

As in most tissues, the eye expresses heme-oxygenases (HO-1 and HO-2), which constitute an essential cytoprotective system that metabolizes heme and generates antioxidants and the bioactive gas carbon monoxide. HO-1 has emerged as a highly inducible and essential cytoprotective gene whose amplification by genetic or pharmacological approaches causes remarkable antiproliferative, anti-inflammatory, and antiapoptotic actions[126,127,128,129,130]. Thus, it is striking that acute and self-resolving corneal injury dramatically up-regulates the resident HO system. The essential role of the HO system in the cornea was established by employing HO-2 knockout mice that failed to induce HO-1 expression in the injured cornea[116,131,132]. In these HO-deficient mice, normal self-resolving inflammation and complete wound closure was shifted to chronic inflammation, failed wound healing, and pronounced corneal neovascularization. Strikingly, the phenotype of chronic injury and inflammatory neovascularization correlated with a 50% reduction in endogenous $LXA_4$ formation. Subsequent studies in mice with an impaired $LXA_4$ biosynthetic pathway (12/15-LOX knockout) demonstrated that induction of HO-1 was impaired and correlated with amplified PMN infiltration, chemokine formation, and delayed wound healing[116]. More importantly, add back experiments with topical $LXA_4$ to the 12/15-LOX knockout mice restored normal HO-1 expression, wound healing, and inflammation. Experiments with human corneal epithelial cells confirmed that specific 15-LOX–derived mediators, $LXA_4$, NPD1, and 17-HDHA, amplify expression of cytoprotective HO-1, a bioactivity which was not shared by 15-HETE[116]. These studies suggest an interdependence of two intrinsic protective circuits in the cornea[116,131], namely HO and the $LXA_4$ circuit. In this regard, it is important to point out that 15-deoxyΔ-$PGJ_2$ and stable analogs of $LXA_4$ have been shown to up-regulate HO-1 expression in macrophages and endothelial cells, respectively[52,53,133].

A protective role for $LXA_4$ has also been identified in intraocular inflammatory diseases, specifically endotoxin-induced uveitis (EIU)[134]. EIU is a common animal model that exhibits many of the key features of human uveitis, including dilation of vessels, macromolecule leakage, edema, and infiltration of leukocytes and lymphocytes. Topical treatment with $LXA_4$ greatly attenuated EIU[134]. The preventative and therapeutic effect of $LXA_4$ on EIU correlated with reduced protein leakage and PMN infiltration into the aqueous humor of the eye, as well as a reduction in proinflammatory mediator (i.e., TNF-α, COX-2, and vascular endothelial growth factor; VEGF) production and NF-κB activation.

A recent report defined the endogenous role of the $LXA_4$ circuit and a $LXA_4$ stable analog in chronic injury–induced inflammatory neovascularization[135,136]. In a model of chronic inflammation (i.e., suture-induced injury), topical treatment with $LXA_4$, but not its metabolic precursor 15-HETE, attenuated formation of blood vessels and reduced expression of critical mediators of inflammatory neovascularization, including key members of the VEGF family (i.e., VEGF-A and the VEGF receptor-3, FLT4)[135]. Treatment with a $LXA_4$ stable analog also reduced the expression of the proinflammatory cytokines TNF-α, IL-1α, and IL-1β[136]. More importantly, pathological angiogenesis correlated with selective up-regulation of 12/15-LOX activity and expression, and regulated expression of $LXA_4$ receptors, which likely is a protective tissue response to counter-regulate nonresolving inflammation. This notion is supported by genetic deletion of 12/15-LOX and 5-LOX, key and obligatory enzymes in the formation of $LXA_4$, respectively[16,100,109], which led to pronounced exacerbation of inflammatory neovascularization coincident with increased expression of VEGF-A and FLT4[135]. Topical treatment with $LXA_4$ rescued 15-LOX knockout mice from exacerbated angiogenesis, providing direct evidence for the $LXA_4$ circuit as an endogenous regulator of pathological angiogenesis in the cornea.

Taken together, well-established and distinct experimental models of inflammation, namely acute, self-resolving inflammation and chronic inflammation with neovascularization, have demonstrated that the endogenous $LXA_4$ circuit has an essential role in limiting the sequelae of injury in the delicate visual axis and interacts with other cytoprotective systems to amplify protective circuits, which may endow ocular tissues such as the cornea with privileged injury responses.

Aside from $LXA_4$, other LOX-derived AA metabolites, namely, 12(S)-HETE and 15(S)-HETE, can exert beneficial actions in the cornea. 12(S)-HETE and 15(S)-HETE can function as intracellular second messengers for growth factors and modulate the proliferation of corneal epithelial cells, thereby promoting wound closure[19,36]. In addition, topically applied 15(S)-HETE increased mucin secretion and protected the cornea from dessicating-induced damage in animal models of dry eye[36]. Although the mechanism of action has not been clearly defined, it has been shown to involve translocation and activation of protein kinase C-α, which is necessary for goblet cell proliferation, thereby increasing mucin secretion[36]. However, the endogenous role of 15(S)-HETE depends on “a where and a when”, as topical application of 15(S)-HETE promotes angiogenesis in the cornea[135] and in human corneal epithelial cells, reduces basal expression of the cytoprotective and anti-inflammatory HO-1[116].

### ω-3 PUFA–Derived Circuits

In addition to AA, cells release other essential PUFAs, including eicosapentaenoic acid (EPA, ω-3 C20:5) and docosahexaenoic acid (DHA, ω-3 C22:6), in response to stress, injury, or inflammatory stimuli (Fig. 3). The importance of essential PUFAs is underscored by the well-established roles of ω-3 PUFAs in normal growth and development, and maintaining human health[137,138,139,140,141,142,143,144]. Moreover, epidemiological studies and clinical trials have demonstrated that consumption of fish oils, which are enriched in EPA and DHA, is cardio- and renoprotective, and lowers the incidence of inflammatory and autoimmune diseases[137,138,142,145,146,147,148,149,150,151,152]. In particular, DHA has been shown to exert anti-inflammatory and immunosuppressive effects[150,153].

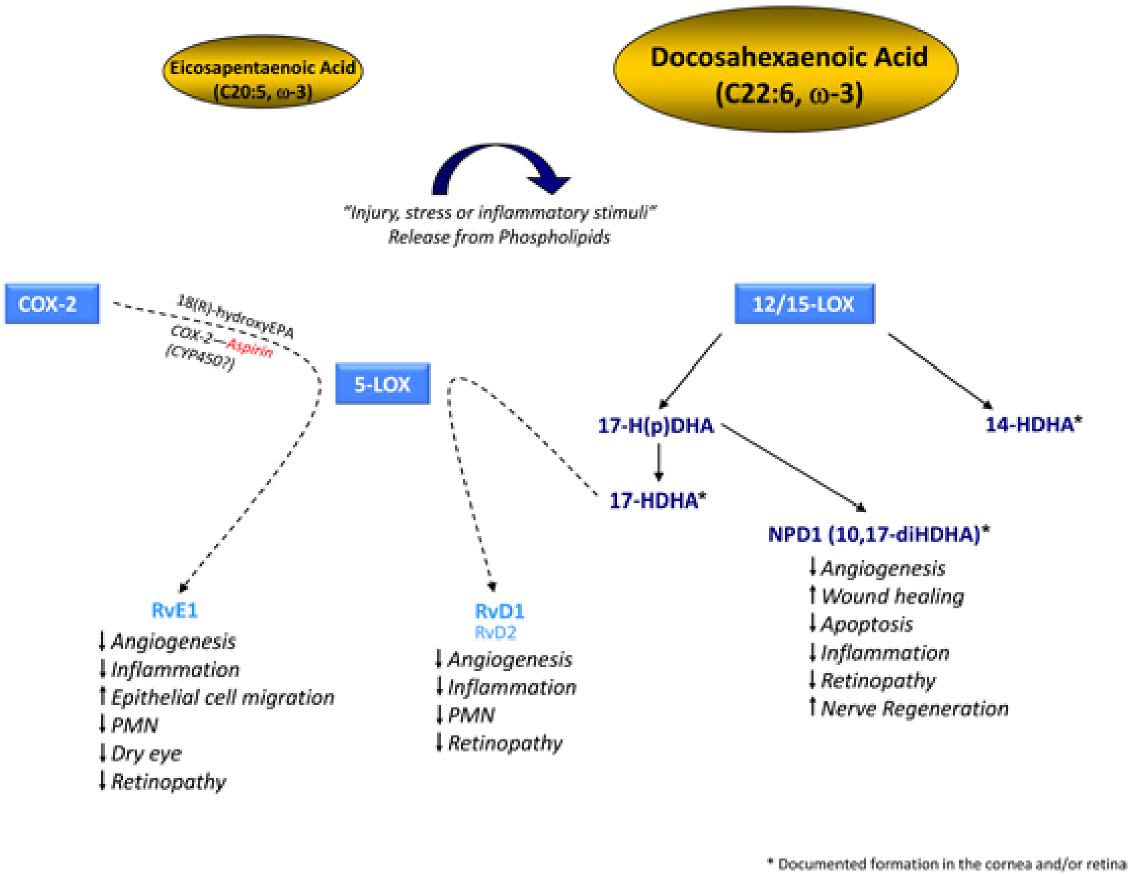

**FIGURE 3.** Protective ω-3 PUFA–derived circuits in ocular inflammation. Following injury, stress, or inflammatory stimuli, ω-3 essential fatty acids, including EPA and DHA, can be released from phospholipid pools to generate LOX-derived resolvins (Rv) and neuroprotectin D1 (NPD1), respectively. These novel EPA- and DHA-derived lipid autacoids exert specific protective actions that control inflammation and pathological angiogenesis, promote epithelial wound healing, and ultimately maintain ocular health. Dark blue font denotes DHA-derived products, light blue font denotes EPA-derived products.

The protective and beneficial effects of dietary ω-3 PUFAs are now recognized as an important factor for ocular health, a notion that is strongly supported by both experimental and clinical data[154,155,156,157,158,159]. In a murine model of macular degeneration, a diet enriched in DHA and EPA reduced the development of retinal lesions[160], and in a murine model of oxygen-induced retinopathy, increasing ω-3 tissue levels by dietary or genetic manipulation reduced pathological retinal neovascularization[161]. Moreover, several studies have shown that infant formulas supplemented with DHA led to an enhancement in visual resolution acuity, maturation of retinal function, and overall neurological performance in preterm and term infants[20,162,163]. Importantly, the therapeutic potential and clinical relevance of supplementing ω-3 intake is exemplified by current clinical trials (i.e., Age-Related Eye Disease Study) that have demonstrated that dietary ω-3 PUFA intake is inversely associated with neovascular age-related macular degeneration (AMD)[164]. Consumption of fish oil–enriched diets containing ω-3 DHA and EPA by persons with moderate-to-high risk for AMD not only lowered the risk of developing neovascular AMD, but also reduced the progression from bilateral drusen to central geographic atrophy over a 12-year period[165,166]. Increased dietary intake of ω-3 PUFAs was also associated with a decreased incidence of dry eye syndrome in a large, well-characterized population of women participating in the Women's Health Study[167]. In parallel, treatment with α-linolenic acid, a precursor for EPA and DHA, reversed the signs and underlying inflammatory changes seen in a murine model of dry eye; leukocyte infiltration into the cornea was reduced, as was corneal and conjunctival expression of TNF-α and IL-1α[168].

The discovery of novel EPA- and DHA-derived lipid autacoids has provided a major breakthrough in understanding the potential molecular mechanism through which these essential ω-3 PUFAs mediate their remarkable beneficial actions[100,101,110,169,170]. These PUFAs can be metabolized, in a manner analogous to LX formation (Figs. 1 and 3), to generate a novel class of immune regulatory and anti-inflammatory protective lipids termed resolvins (Rv), so named because they were first identified as resolution-phase interaction products. Based on the substrate, they belong to the RvE series if derived from EPA or the RvD series if derived from DHA[100,101,171,172]. In addition to resolvins, endogenous formation of another DHA-derived lipid mediator, 10,17(S)-docosatriene, has been identified in both humans and rodents[169,173]. 10(R),17(S)-docosatriene has been termed neuroprotectin D1 (NPD1) if generated in neural tissue and based on its protective actions in neurons, glial cells, stroke, and animal models of Alzheimer's disease; the term protectin D1 (PD1) has been used if the bioactivity and endogenous formation are outside the nervous system[20,100,101,154,171,174,175]. NPD1 is of particular interest since its biosynthetic pathway, unlike that of resolvins and LXs, is triggered by a single LOX enzyme (Fig. 3), namely, 15-LOX, which is highly expressed in epithelial cells of both the cornea and retina. Data from 12/15-LOX knockout mice and siRNA targeting human 15-LOX have identified ALOX15 as a key enzyme for the formation of NPD1. The complete structure of NPD1, RvD1, RvE1, and RvD2 have recently been disclosed[176,177,178,179]. These EPA- and DHA-derived lipid autacoids exhibit anti-inflammatory and proresolving bioactivity, which are stereoselective and evoked in the pico- to nanomolar range. The protective ω-3–derived signals have distinct, but overlapping, bioactivity and both *in vitro* and *in vivo* experiments have demonstrated that similar to $LXA_4$, resolvins and protectins reduce leukocyte infiltration, regulate cytokines/chemokines and reactive oxygen species, and increase macrophage ingestion of apoptotic PMNs[20,98,146,169,171].

It is important to recognize that without marked dietary supplementation, EPA concentrations in human tissues, plasma, and milk are very low. By contrast, DHA, like AA, is found in all human tissues, plasma, and milk at concentrations 1–20% of total fatty acids[180]. Moreover, in the cerebral cortex, sperm, and retina, DHA is present at higher concentrations than AA[180]. In particular, retinal photoreceptor outer segments have the highest DHA content of any cell[181,182,183] and unusual DHA-retention ability[184,185,186]. Prolonged dietary deprivation of ω-3 PUFAs has been shown to correlate with severe visual impairment[187,188]. The tissue levels of DHA exceed those of EPA five- to 30 fold in most tissues and more than 100-fold in the retina and central nervous system, which is consistent with the fact that human metabolic pathways for all dietary ω-3 PUFAs (α-linolenic acid [C18:3] or EPA) preferentially lead to the formation of DHA. However, endogenous formation of DHA from ω-3 PUFA

precursors, such as α-linolenic acid, is very inefficient. It is now recognized that the major source of DHA is direct consumption, which truly places DHA as an essential fatty acid. Hence, it is not surprising that several lines of evidence illustrate a critical role for DHA in human physiology, namely, neuronal and retinal photoreceptor functions[20,174,186,189]. Indeed, experimental evidence demonstrates decreased blood levels of DHA in various forms of retinitis pigmentosa and in animal models of inherited retinal degeneration[20].

Like the cornea, the subretinal space is an immune-privileged site and the retinal pigmented epithelial cells (RPE) have an important role in maintaining the immune privilege[190]. RPE are highly specialized, and are essential to maintain and phagocytose the photoreceptors (rods and cones), recycle DHA back to the inner segments during photoreceptor outer segment renewal, and form the retinal-blood barrier. Failure of RPE cells to function properly leads to photoreceptor damage or death, and, consequently, impaired vision and eventual blindness. The photoreceptors and RPE compose a region of the retina that is particularly prone to oxidative stress. As increased oxidative stress, trauma, or retinal detachment induces RPE dysfunctions that can contribute to neovascularization and subsequently AMD or other retinal degenerations, RPE have developed endogenous mechanisms to counteract these challenges[20,154,174,175,191]. In this regard, DHA, which is enriched in these cells, exerts a striking protective effect. When confronted with excessive oxidative stress, RPE cells increase production of DHA-derived NPD1[192]. NPD1 inhibits oxidative stress–mediated proinflammatory gene induction and apoptosis, and, consequently, promotes RPE cell survival[193]. Further, human RPE cells deficient in 15-LOX type 1 exhibit increased sensitivity to oxidative stress–induced apoptosis, which was rescued selectively by NPD1, but not other 15-LOX type 1–derived metabolites (i.e., 12(S)-HETE, 15(S)-HETE)[77]. NPD1 has also been shown to inhibit retinal ganglion cell apoptosis following optic nerve transaction[60], as well as inhibit pathological retinal angiogenesis along with RvD1 and RvE1, an effect mediated in part through suppression of the inflammatory cytokine TNF-α[161]. In a recent report, RvD1 and RvE1 were also shown to reduce inflammatory signaling and PMN migration in choroid-retinal endothelial cells *in vitro*[194].

Recent reports also provide evidence for a role of ω-3–derived lipid autacoids in the resolution of immune responses in the cornea. Importantly, endogenous formation of NPD1 and 17(S)-HDHA was first documented in both healthy and injured corneas obtained from mice fed an ω-3–enriched diet[76]. Similar to the protective bioactivity of $LXA_4$ following de-epithelialization, topical treatment with NPD1 accelerated wound healing without impairing PMN recruitment[76]. In a scratch wound assay, RvE1 and resolvin analogs were shown to stimulate human corneal epithelial cell migration via activation of phosphoinositide 3-kinase and p38 mitogen activated protein kinase signaling, thereby accelerating wound healing[195,196]. Resolvin analogs also exert beneficial effects in dry eye; in a mouse model of dry eye, topical application of RvE1 analogs increased tear production as well as epithelial cell density, suggesting that resolvins could play a role in maintaining the integrity of epithelia damaged by dry eye[197]. Expression of COX-2 and α-smooth muscle actin was also decreased, as was macrophage infiltration. Hence, it is likely that the protective actions of ω-3–derived lipid autacoids in dry eye are multifactorial, and involve modulation of the inflammatory and immune components. Indeed, RVE1 has been shown to inhibit PMN infiltration, regulate T-helper cell survival and differentiation, and regulate macrophage function and cytokine expression in animal models of acute and allergic inflammation[100,198,199]. In addition, RvD1 and RvE1 exert antiangiogenic effects in models of corneal neovascularization induced by suture or micropellets containing IL-1β or VEGF-A, which was associated with reducing leukocyte infiltration and the gene expression of inflammatory cytokines[136].

Maintenance of a healthy corneal surface is in part dependent on the integrity of corneal sensory nerves. Indeed, the impairment of corneal nerves disrupts the corneal reflex arc, affecting lacrimal gland function and subsequently tear production, causing dry eye[200]. Interestingly, recent studies now implicate a role for NPD1 in the regeneration of corneal nerves damaged after surgery[201,202]. Following photorefractive keratectomy, administration of DHA with nerve growth factor significantly increased nerve density and, more importantly, increased epithelial cell proliferation, demonstrating a healthy epithelium[202]. In subsequent studies, treatment with DHA and pigment epithelial-derived growth factor following lamellar keratectomy also promoted corneal nerve regeneration, which was associated with synthesis of NPD1[201]. Taken together, these studies demonstrate a role for DHA-derived autacoids, namely, NPD1, in regenerating corneal innervation after refractive surgery, thereby repairing and protecting the ocular surface, and preventing the development of dry eye and neurotrophic keratopathies[200].

### CONCLUSION

Inflammation is an essential and beneficial response to tissue insult, injury, hypoxia, and infection. A tightly regulated inflammatory program, and its successful execution and resolution, are particularly critical and highly evolved in the delicate visual axis, as uncontrolled ocular inflammation and disordered wound repair can lead to blindness. It is now appreciated that acute inflammation is a precarious innate response whose successful outcome is dependent on the balanced formation of pro- and anti-inflammatory lipid autacoids, the active resolution of leukocytes, and restoration of homeostasis. In contrast to most tissues/organs, our understanding of lipid mediator circuits in the eye is relatively unexplored. A rapidly evolving body of work provides strong evidence for protective lipid circuits as key components of the ocular inflammatory/reparative response. Specifically, 15-LOX–derived autacoids, such as the eicosanoid $LXA_4$ and DHA-derived NPD1, have emerged as central components of an intrinsic and essential protective pathway in the retina and cornea. The regulation and mechanisms of action for these intrinsic and protective lipid circuits remain to be clearly defined, and are of great clinical interest for the delicate visual axis as therapeutic targets and as a potential underlying cause of ocular diseases.

### ACKNOWLEDGMENTS

We gratefully acknowledge grants from the National Institutes of Health, EY016136 and P30EY003176, for supporting research in the author’s laboratory (K.G.), which is cited in this review.
